# FGF-23, Left Ventricular Hypertrophy, and Mortality in Patients With CKD: A Revisit With Mediation Analysis

**DOI:** 10.1016/j.jacadv.2023.100747

**Published:** 2023-12-09

**Authors:** Naoko Hidaka, Kosuke Inoue, Hajime Kato, Yoshitomo Hoshino, Minae Koga, Yuka Kinoshita, Yuichi Takashi, Noriko Makita, Seiji Fukumoto, Masaomi Nangaku, Nobuaki Ito

**Affiliations:** a Division of Nephrology and Endocrinology, The University of Tokyo Hospital, Bunkyo, Tokyo, Japan; b Osteoporosis Center, The University of Tokyo Hospital, Bunkyo, Tokyo, Japan; c Department of Social Epidemiology, Graduate School of Medicine, Kyoto University, Sakyo, Kyoto, Japan; d Department of Endocrinology and Diabetes Mellitus, Fukuoka University School of Medicine, Jonan, Fukuoka, Japan; e Fujii Memorial Institute of Medical Sciences, Institute of Advanced Medical Sciences, Tokushima University, Tokushima, Japan

**Keywords:** all-cause mortality, cardiovascular disease, chronic kidney disease, fibroblast growth factor-23, left ventricular hypertrophy, mediation analysis

## Abstract

**Background:**

In patients with chronic kidney disease (CKD), fibroblast growth factor (FGF)-23 is suspected to cause death or cardiovascular disease by inducing left ventricular hypertrophy (LVH).

**Objectives:**

This study aims to quantify the mediational effect of LVH in the hypothetical causal pathway from FGF-23 to long-term adverse outcomes.

**Methods:**

From 3,939 adults with CKD stages 2 to 4 enrolled in the CRIC (Chronic Renal Insufficiency Cohort) study, 2,368 participants with available data of FGF-23, left ventricular mass index at 1 year, and covariates were included. We employed linear and Cox proportional hazards regression models to investigate the association between FGF-23 and LVH, all-cause mortality, atrial fibrillation (AF), or congestive heart failure (CHF). Mediation analysis was used within a counterfactual framework to decompose the effect of FGF-23 into natural direct and indirect effects.

**Results:**

Among 2,368 participants (mean age: 57.7 years, 1,252 males, median FGF-23 level: 138.8 RU/mL), left ventricular mass index was positively correlated with FGF-23. During a median of 12.0, 11.1, and 11.1 years, FGF-23 was associated with all-cause mortality (HR: 1.62, 95% CI: 1.24-2.12), AF (HR: 1.58, 95% CI: 1.12-2.24), and CHF (HR: 1.32, 95% CI: 0.95-1.84) when the highest quartile was compared to the lowest quartile. LVH mediated 7.4%, 11.2%, and 21.9% of the effect of FGF-23 on all-cause mortality, AF, and CHF, respectively.

**Conclusions:**

In CKD patients, FGF-23 had a minor effect on the development of long-term adverse outcomes through LVH. Other potential mediators and the validity of negative effect of FGF-23 should be explored.

Fibroblast growth factor (FGF)-23 is a phosphate-regulating hormone that is principally secreted from osteoblasts and mature osteocytes.^1^, ^2^, ^3^ Upon binding to the heterodimeric complex of FGF receptor 1 and alpha Klotho expressed in the renal tubule, FGF-23 downregulates the expression of sodium-phosphate cotransporters and decreases active 1,25-hydroxyvitamin D by downregulating 1 alpha-hydroxylase and upregulating 24-hydroxylase.^1^^,^^2^^,^^4^ Overall, FGF-23 facilitates the excretion of phosphate in the tubule and indirectly suppresses the absorption of phosphate in the intestine. Because the kidney plays a pivotal role in modulating serum phosphate levels, the secretion and serum levels of FGF-23 are physiologically upregulated to counteract the increased load of phosphate in patients with chronic kidney disease (CKD). In CKD stage 3, FGF-23 starts to rise exponentially as the estimated glomerular filtration rate (eGFR) declines.^5^

When FGF-23 was first identified as the causative molecule for the development of autosomal dominant hypophosphatemic rickets/osteomalacia and tumor-induced osteomalacia, it was conceived as the molecule dedicated solely to the regulation of serum phosphate without other function.^6^^,^^7^ Subsequently, in 2008, FGF-23 measured by a C-terminal FGF-23 assay (C-term FGF-23) was reported as a sensitive and independent marker to predict mortality in CKD patients who were beginning hemodialysis, which implied the direct effect of FGF-23 on mortality among CKD patients.^8^ In 2011, the associations between elevated C-term FGF-23 and increased risk of all-cause death or left ventricular hypertrophy (LVH) among patients with CKD stages G2 to G4 were revealed with data from the CRIC (Chronic Renal Insufficiency Cohort) study,^9^^,^^10^ while the major cause of death in CKD patients is cardiovascular disease, and LVH in CKD has been reported as a negative prognostic factor.^11^ The in vivo study to confirm the effect of elevated FGF-23 on the heart was conducted by Faul et al^10^ in 2011, which described that FGF-23 has the potential to induce cardiomyocyte hypertrophy in a Klotho-independent manner. In this report, intramyocardial or intravenous injection of FGF-23 induced LVH in wild-type mice, and the development of LVH was attenuated by FGF receptor blockade in CKD model mice.

However, there are some controversies regarding the hypothesis that FGF-23 is the direct stimulant to the heart contributing to the development of LVH. Shalhoub et al^12^ reported the lack of effect of FGF-23 antagonizing antibody on increased expression of cardiac hypertrophy markers in 5/6 nephrectomized rats. In addition, we reported that 24 patients with FGF-23-related hypophosphatemic diseases did not present LVH, and serum FGF-23 measured by intact FGF-23 assay was not associated with parameters of cardiac dysfunction or atherosclerosis in 119 hemodialysis patients.^13^^,^^14^ Therefore, it is still unknown whether FGF-23 is just a surrogate marker for CKD patients with a high risk of death and cardiovascular outcomes or a direct genuine negative effector on the heart.

On another front, some factors that upregulate the transcription of *FGF23* have been reported recently, including inflammatory cytokines,^15^^,^^16^ iron deficiency,^17^^,^^18^ parathyroid hormone (PTH),^19^^,^^20^ and erythropoietin.^21^^,^^22^ It is possible that these factors acted as confounders in the association between C-term FGF-23 and long-term adverse health outcomes, although the previous results derived from the CRIC study were not adjusted for all of these factors.^9^^,^^10^^,^^23^, ^24^, ^25^

Here, using the multiracial, prospective, and observational CKD cohort from the CRIC study,^26^ we investigated the risks of all-cause mortality and cardiovascular outcomes (atrial fibrillation [AF] and congestive heart failure [CHF]) for a longer observation period than those in previous reports,^9^^,^^24^^,^^25^ with additional adjustment for recently identified modifiers of C-term FGF-23. Furthermore, we conducted a mediation analysis to examine the hypothetical causal pathway by which C-term FGF-23 increases all-cause mortality and cardiovascular outcomes through increased left ventricular mass index (LVMI).

## Methods

### Data sources and study population

The CRIC study is an observational prospective cohort study to examine the risk factors for the progression of CKD and cardiovascular disease among multiracial participants with CKD stages 2 to 4.^26^ Between April 2003 and September 2008 at 7 institutions in the United States, phase 1 of the CRIC study enrolled 3,939 adults aged 21 to 74 years with an eGFR between 20 and 70 mL/min/1.73 m^2^. Participants underwent extensive clinical evaluation at baseline and annual clinic visits, 6 months after which they were interviewed by telephone. All participants provided written informed consent, the form of which was approved at each clinical center according to the guidelines of their institutional review board.

### FGF-23 assay

Baseline plasma C-term FGF-23 was measured in samples from 3,879 of the 3,939 participants using a C-terminal assay (Immutopics).^5^

### Left ventricular mass index

Transthoracic echocardiography was scheduled for all participants 1 year after the baseline visit. Left ventricular mass was derived from 2-dimensional images, and LVMI was calculated by the following formula: LVMI = (left ventricular mass)/(height [meters]).^2^^,^^7^ LVH was defined as LVMI >50 g/m^2.7^ for males and LVMI >47 g/m^2.7^ for females.

### All-cause mortality, AF, and CHF

Outcomes included all-cause mortality, the incidence of AF, and the incidence of CHF through December 31, 2018. Death was ascertained through reports from the subject’s proxies or other contacts, primary care physicians, or the database of death certificates in the United States (National Death Index). AF events included both AF and atrial flutter, which were reviewed by relevant medical records and electrocardiograms. CHF was identified clinically by the presence of a syndrome characterized by breathlessness, pulmonary congestion, effort intolerance, fluid retention, or peripheral hypoperfusion. Clinical symptoms, radiographic evidence of pulmonary edema, physical examination of the heart and lungs, invasive hemodynamic monitoring data, and echocardiographic findings were assessed according to Framingham Heart Study clinical criteria.^25^^,^^27^ CHF events were classified into definite and probable statuses by 2 independent reviewers, both of which were considered CHF events in the current analysis.

### Other covariates

Demographic data, physical measurements, laboratory samples, past medical history of the participants and families, and medications were collected at the baseline visit. PTH was previously measured with a total PTH assay, which detected the 1 to 84 PTH molecule and 7 to 84 fragments (Scantibodies Laboratory, Inc).^5^ N-terminal pro-B-type natriuretic peptide (NT-proBNP) was measured by the Elecsys 2010 analyzer (Roche Diagnostics Corporation).^28^ Tumor necrosis factor-α, interleukin (IL)-6, and IL-1β were measured with high-sensitivity sandwich enzyme-linked immunosorbent assays (Quantikine HS, R&D systems, Inc). High-sensitivity C-reactive protein and fibrinogen were measured using specific laser-based immunonephelometric methods on a BN II (Siemens Healthcare Diagnostics Inc).^29^

### Statistical analysis

First, we used multivariable linear regression models to examine the relationship between C-term FGF-23 level and natural log-transformed LVMI: log (LVMI). In Model 1, we included demographic factors (age, sex, race, and ethnicity). Following a prior study,^9^ in Model 2, we adjusted for covariates in Model 1 plus body mass index, current smoking, systolic blood pressure, history of coronary artery disease, CHF, stroke, peripheral vascular disease, and diabetes, eGFR based on creatinine, age, sex, and race (the modified Modification of Diet in Renal Disease study equation),^30^ natural log-transformed urine albumin-to-creatinine ratio, serum albumin, hemoglobin, serum calcium, serum phosphate, natural log-transformed total PTH, low-density lipoprotein, and medication use (beta-blockers, angiotensin-converting enzyme inhibitors or angiotensin II receptor blockers, aspirin, and statins). Model 3 included the covariates in Model 2 plus family history of coronary artery disease, NT-proBNP, tumor necrosis factor-α, fibrinogen, high-sensitivity C-reactive protein, IL-6, IL-1β, and medication use (diuretics, active vitamin D, phosphate binders, and erythropoiesis-stimulating agents).

Second, we used Cox proportional hazards regression models to examine the relationships of C-term FGF-23 with all-cause mortality, incident AF, and incident CHF, adjusted for the abovementioned covariates included in linear regression models. In all models, C-term FGF-23 was analyzed in the form of categorical variables. Considering the simultaneous risk for incident AF and CHF, we also carried out a competing-risk analysis, as suggested by Fine and Gray,^31^ where we determined the subdistribution hazard by creating risk sets that encompassed individuals without any event as well as those who experienced competing events such as death.

Third, we employed causal mediation analysis within the counterfactual framework to decompose the total effects into the direct effect and the indirect effect using the R package “CMAverse”.^32^ The direct effect refers to the effect of C-term FGF-23 levels on outcomes without going through increased LVMI. The indirect effect represented the effects of C-term FGF-23 levels on outcomes mediated through increased LVMI. In these mediation analyses, the same variables as in Model 3 described above were adjusted. Total effect, direct and indirect effects, and proportion mediated were calculated according to the quartiles of C-term FGF-23 level, with the lowest quartile as the reference group. Our mediation analyses followed a recent guideline (AGReMA Statement).^33^ Detailed steps and required assumptions for causal mediation analysis are described in the Supplemental Methods.

We conducted several sensitivity analyses. First, given the feedback loop between serum FGF-23 and phosphate levels, we conducted our analyses without adjustment for the relevant biomarkers (ie, serum calcium, serum phosphate, total PTH, and use of active vitamin D and phosphate binders). Second, as cystatin C is less affected by muscle mass and diet, we analyzed the data using eGFR based on cystatin C instead of glomerular filtration rate based on creatinine.^34^ Third, we reconducted the mediation analysis using the same covariates included in a previous study (Model 2).^9^ Last, the analyses restricted to “definite” CHF were repeated.

To estimate the robust 95% CIs, we repeated the analyses on 1,000 bootstrapped samples, which involved resampling the original data with replacement to create multiple datasets for evaluating the stability of the results.^35^ The estimated 95% CIs were not adjusted for multiple comparisons and thus need to be interpreted with caution. All statistical analyses were conducted by R software version 4.3.0 or Stata version 16.

## Results

Among 3,879 participants whose baseline plasma C-term FGF-23 levels had been measured, 84 participants died within a year, and then LVMIs were available for 2,610 participants from echocardiography at 1 year after the baseline visit. A total of 242 participants with missing data on covariates were excluded (Supplemental Table 1), resulting in a final analytical sample of 2,368 participants.

Among 2,368 participants, the mean age was 57.7 ± 10.7 years; 1,252 participants (52.9%) were male; and White and Black people accounted for approximately 90% of all participants (Table 1). The median level of baseline C-term FGF-23 was 138.8 RU/mL.

**Table 1:** Baseline Participant Characteristics According to Quartiles of C-Term FGF-23 Level

	All Participants (N = 2,368)	Quartiles of C-Term FGF-23 Level			
		Q1 (Lowest) <10-93.2 (n = 592)	Q2 93.4-138.7 (n = 592)	Q3 139.0-221.0 (n = 592)	Q4 (Highest) 221.6-14,318.9 (n = 592)
Characteristics					
Age, y	57.7 ± 10.7	55.6 ± 11.3	58.9 ± 10.1	58.3 ± 11.0	58.1 ± 10.2
Male	1,252 (52.9)	361 (61.0)	331 (55.9)	318 (53.7)	242 (40.9)
Race					
White	1,093 (46.2)	296 (50.0)	290 (49.0)	271 (45.8)	236 (39.9)
Black	1,054 (44.5)	254 (42.9)	241 (40.7)	256 (43.2)	303 (51.2)
Hispanic	117 (4.9)	12 (2.0)	32 (5.4)	35 (5.9)	38 (6.4)
Others	104 (4.4)	30 (5.1)	29 (4.9)	30 (5.1)	15 (2.5)
Body mass index, kg/m^2^	31.7 ± 7.3	30.3 ± 6.3	31.3 ± 7.1	31.7 ± 7.2	33.4 ± 8.3
Current smoker	283 (12.0)	35 (5.9)	51 (8.6)	81 (13.7)	116 (19.6)
Systolic blood pressure, mm Hg	126.7 ± 21.1	122.3 ± 18.9	124.6 ± 19.9	129.3 ± 22.0	130.7 ± 22.2
Past history					
Coronary artery disease	493 (20.8)	83 (14.0)	128 (21.6)	136 (23.0)	146 (24.7)
Congestive heart failure	212 (9.0)	22 (3.7)	29 (4.9)	54 (9.1)	107 (18.1)
Stroke	220 (9.3)	45 (7.6)	41 (6.9)	60 (10.1)	74 (12.5)
Peripheral vascular disease	142 (6.0)	19 (3.2)	27 (4.6)	37 (6.3)	59 (10.0)
Diabetes	1,068 (45.1)	163 (27.5)	248 (41.9)	306 (51.7)	351 (59.3)
Family history of coronary artery disease	352 (14.9)	71 (12.0)	80 (13.5)	97 (16.4)	104 (17.6)
Laboratory results					
Estimated glomerular filtration rate, mL/min/1.73 m^2^	43.5 ± 13.2	52.7 ± 11.9	46.1 ± 11.3	40.5 ± 10.3	34.7 ± 11.9
Urine albumin-to-creatinine ratio, mg/g	509.9 ± 1,376.5	199.8 ± 639.4	286.2 ± 726.1	549.1 ± 1,274.2	1,004.2 ± 2,153.1
Serum albumin, g/dL	4.0 ± 0.4	4.1 ± 0.4	4.0 ± 0.4	4.0 ± 0.4	3.8 ± 0.5
Hemoglobin, g/dL	12.7 ± 1.8	13.5 ± 1.6	12.9 ± 1.6	12.5 ± 1.5	11.8 ± 1.8
Calcium, mg/dL	9.2 ± 0.5	9.2 ± 0.4	9.3 ± 0.5	9.2 ± 0.5	9.2 ± 0.6
Phosphate, mg/dL	3.7 ± 0.7	3.4 ± 0.5	3.6 ± 0.6	3.7 ± 0.6	4.0 ± 0.8
Total parathyroid hormone, pg/mL	71.5 ± 67.2	47.3 ± 31.9	57.8 ± 39.6	74.0 ± 52.4	106.9 ± 103.5
N-terminal pro-B-type natriuretic peptide, pg/mL	440.8 ± 1,484.1	155.4 ± 281.7	228.3 ± 391.4	416.0 ± 1,145.3	963.3 ± 2,622.4
LDL, mg/dL	102 ± 35	107 ± 33	103 ± 32	101 ± 37	98 ± 37
Tumor necrosis factor-α, pg/mL	3.1 ± 12.6	2.3 ± 5.2	2.5 ± 4.4	3.9 ± 19.1	3.9 ± 15.0
Fibrinogen, g/L	4.1 ± 1.2	3.7 ± 0.9	3.9 ± 1.0	4.2 ± 1.2	4.6 ± 1.3
High sensitivity C-reactive protein, mg/L	5.3 ± 9.6	4.4 ± 7.6	4.2 ± 6.4	5.0 ± 7.5	7.6 ± 14.4
Interleukin-6, pg/mL	4.1 ± 17.8	2.8 ± 16.9	2.9 ± 13.3	4.1 ± 16.4	6.7 ± 23.0
Interleukin-1β, pg/mL	1.1 ± 3.3	0.8 ± 2.2	1.0 ± 2.5	1.3 ± 4.5	1.5 ± 3.6
Medication use					
Beta-blockers	1,132 (47.8)	208 (35.1)	261 (44.1)	305 (51.5)	358 (60.5)
Angiotensin-converting enzyme inhibitors or angiotensin-II receptor blockers	1,608 (67.9)	349 (59.0)	425 (71.8)	428 (72.3)	406 (68.6)
Diuretics	1,363 (57.6)	236 (39.9)	329 (55.6)	354 (59.8)	444 (75.0)
Aspirin	1,037 (43.8)	226 (38.2)	265 (44.8)	261 (44.1)	285 (48.1)
Active vitamin D	75 (3.2)	6 (1.0)	9 (1.5)	24 (4.1)	36 (6.1)
Phosphate binders	161 (6.8)	28 (4.7)	40 (6.8)	38 (6.4)	55 (9.3)
Erythropoiesis-stimulating agents	96 (4.1)	7 (1.2)	15 (2.5)	24 (4.1)	50 (8.4)
Statins	1,334 (56.3)	266 (44.9)	331 (55.9)	367 (62.0)	370 (62.5)

Values are mean ± SD or n (%).

FGF = fibroblast growth factor.

### FGF-23 and LVH

The mean LVMI was 51.3 g/m^2.7^, and 1,199 participants (50.6%, 598 males and 601 females) had LVH at 1 year after the baseline visit. When we categorized participants into quartiles based on C-term FGF-23 levels, the mean LVMI was 46.8 g/m^2.7^ for Q1 (the lowest quartile), 49.3 g/m^2.7^ for Q2, 51.6 g/m^2.7^ for Q3, and 57.4 g/m^2.7^ for Q4 (the highest quartile). In the parsimonious model adjusting for age, sex, and race/ethnicity (Model 1), we found an association between higher quartiles of C-term FGF-23 and higher log(LVMI), and the magnitude of association was attenuated when we additionally adjusted for covariates based on a previous study^9^ (Model 2) (Table 2). In our fully adjusted regression model (Model 3), the association with log (LVMI) with statistical significance was found for Q4 (β = 3.22 × 10^−2^; 95% CI: 0.30 × 10^−2^ to 6.14 × 10^−2^) but not for Q2 or Q3 compared to Q1, meaning that the Q4 group had around 1.03-fold increase in LVMI (calculated from fitted values of LVMI from the regression model) compared to the Q1 group.

**Table 2:** Baseline C-Term FGF-23 Levels and Left Ventricular Hypertrophy at the 1-Year Follow-Up

log (LVMI)	Model 1^a^		Model 2^b^		Model 3^c^	
	β × 10^2^	95% CI	β × 10^2^	95% CI	β × 10^2^	95% CI
Quartiles of FGF-23						
Q1 (the lowest)	Reference		Reference		Reference	
Q2	4.27	1.52-7.03	−0.16	−2.56 to 2.24	−0.37	−2.77 to 2.02
Q3	8.39	5.64-11.15	−0.47	−3.03 to 2.10	−0.52	−3.08 to 2.04
Q4 (the highest)	18.57	15.78-21.35	3.77	0.85-6.68	3.22	0.30-6.14
*P* for trend	<0.001		0.013		0.031	

FGF = fibroblast growth factor; LVMI = left ventricular mass index.

a Adjusted for age, sex, race and ethnicity.

b Adjusted for age, sex, race and ethnicity, body mass index, current smoking, systolic blood pressure, history of coronary artery disease, congestive heart failure, stroke, peripheral vascular disease, diabetes, estimated glomerular filtration rate, natural log-transformed urine albumin-to-creatinine ratio, serum albumin, hemoglobin, serum calcium, serum phosphate, natural log-transformed total parathyroid hormone, low-density lipoprotein, and medication use (beta-blockers, angiotensin-converting enzyme inhibitors or angiotensin II receptor blockers, aspirin, and statins).

c Adjusted for covariates in Model 2 plus family history of coronary artery disease, N-terminal pro-B-type natriuretic peptide, tumor necrosis factor α, fibrinogen, high sensitivity C-reactive protein, interleukin-6, interleukin-1β, and medication use (diuretics, active vitamin D, phosphate binders, and erythropoiesis-stimulating agents).

### FGF-23 and all-cause mortality, AF, and CHF

The median duration of follow-up until mortality ascertainment was 12.0 (IQR: 8.8-13.2) years. During this period, 776 participants (32.8%) died from all causes. AF and CHF were identified in 418 (17.7%) and 506 (21.4%, including 108 “probable” events) participants over the median of 11.1 (IQR: 6.1-12.8) years and 11.1 (IQR: 5.6-12.8) years of follow-up, respectively. Their incidence rates were 1.87 (95% CI: 1.69-2.05) per 100 patient-years for AF and 2.30 (95% CI: 2.10-2.50) per 100 patient-years for CHF. Across all models, we found the tendency that the higher quartiles of C-term FGF-23 had higher HRs for all-cause mortality, incident AF, and incident CHF (Table 3). In the fully adjusted multivariable Cox hazard regression model (Model 3), we found an increased risk of all-cause mortality (HR: 1.62; 95% CI: 1.24-2.12), incident AF (HR: 1.58; 95% CI: 1.12-2.24), and incident CHF (HR: 1.32; 95% CI: 0.95-1.84) for Q4 compared to Q1. The results were consistent for incident AF and incident CHF when implementing a competing-risk regression based on Fine and Gray's proportional subhazards model (Supplemental Table 2).

**Table 3:** Baseline C-Term FGF-23 Levels and All-Cause Mortality, Incidence of Atrial Fibrillation or Congestive Heart Failure

	Quartiles of FGF-23	Unadjusted		Model 1^a^		Model 2^b^		Model 3^c^	
		HR	95% CI	HR	95% CI	HR	95% CI	HR	95% CI
All-cause mortality	Q1 (the lowest)	Reference		Reference		Reference		Reference	
	Q2	1.23	0.96-1.59	1.13	0.88-1.46	0.90	0.70-1.17	0.90	0.70-1.17
	Q3	2.21	1.76-2.77	2.10	1.67-2.65	1.29	1.01-1.65	1.30	1.01-1.67
	Q4 (the highest)	3.70	2.98-4.60	3.86	3.09-4.81	1.71	1.31-2.22	1.62	1.24-2.12
	*P* for trend	<0.001		<0.001		<0.001		<0.001	
Atrial fibrillation	Q1 (the lowest)	Reference		Reference		Reference		Reference	
	Q2	1.26	0.93-1.71	1.14	0.84-1.55	1.02	0.74-1.40	1.03	0.75-1.41
	Q3	1.67	1.25-2.25	1.60	1.19-2.15	1.18	0.85-1.62	1.19	0.86-1.64
	Q4 (the highest)	2.51	1.89-3.32	2.69	2.01-3.58	1.66	1.18-2.35	1.58	1.12-2.24
	*P* for trend	<0.001		<0.001		<0.01		<0.01	
Congestive heart failure	Q1 (the lowest)	Reference		Reference		Reference		Reference	
	Q2	1.50	1.10-2.05	1.39	1.01-1.90	1.00	0.72-1.37	0.99	0.72-1.36
	Q3	2.25	1.67-3.01	2.12	1.57-2.85	1.01	0.73-1.39	0.99	0.71-1.36
	Q4 (the highest)	4.34	3.30-5.72	4.24	3.21-5.62	1.37	0.98-1.90	1.32	0.95-1.84
	*P* for trend	<0.001		<0.001		0.062		0.096	

FGF = fibroblast growth factor.

a Adjusted for age, sex, race and ethnicity.

b Adjusted for age, sex, race and ethnicity, body mass index, current smoking, systolic blood pressure, history of coronary artery disease, congestive heart failure, stroke, peripheral vascular disease, and diabetes, estimated glomerular filtration rate, natural log-transformed urine albumin-to-creatinine ratio, serum albumin, hemoglobin, serum calcium, serum phosphate, natural log-transformed total parathyroid hormone, low-density lipoprotein, and medication use (beta-blockers, angiotensin-converting enzyme inhibitors or angiotensin II receptor blockers, aspirin, and statins).

c Adjusted for covariates in Model 2 plus family history of coronary artery disease, N-terminal pro-B-type natriuretic peptide, tumor necrosis factor α, fibrinogen, high sensitivity C-reactive protein, interleukin-6, interleukin-1β, and medication use (diuretics, active vitamin D, phosphate binders, and erythropoiesis-stimulating agents).

### FGF-23 and long-term adverse health outcomes through increased LVMI

In our causal mediation analyses, we estimated that increased LVMI mediated 7.40% of the association between C-term FGF-23 (Q4 vs Q1) and all-cause mortality (total effect: HR: 1.69; 95% CI: 1.27-2.24; indirect effect: HR: 1.03; 95% CI: 1.00-1.07) and 11.21% of the association between C-term FGF-23 (Q4 vs Q1) and incident AF (total effect: HR: 1.68; 95% CI: 1.17-2.46; indirect effect: HR: 1.05; 95% CI: 1.00-1.10) (Table 4, Central Illustration). We also estimated 21.85% mediation of the increased LVMI in the association between C-term FGF-23 and incident CHF, but the 95% CI of the total effect included 1 (total effect: HR: 1.42; 95% CI: 0.99-2.01; indirect effect: HR: 1.07; 95% CI: 1.00-1.15) (Table 4, Central Illustration).

**Table 4:** Direct and Indirect Effects of C-Term FGF-23 Levels on All-Cause Mortality and the Incidence of Atrial Fibrillation or Congestive Heart Failure via Left Ventricular Hypertrophy^a^

	Quartiles of FGF-23	Total Effect (TE)	Direct Effect (DE)	Indirect Effect (IE)	% Mediated^b^
All-cause mortality	Q1 (the lowest)	Reference	Reference	Reference	Reference
	Q2	0.89 (0.68-1.15)	0.89 (0.69-1.16)	1.00 (0.97-1.02)	NA
	Q3	1.28 (0.97-1.66)	1.29 (0.98-1.67)	1.00 (0.97-1.02)	NA
	Q4 (the highest)	1.69 (1.27-2.24)	1.63 (1.23-2.19)	1.03 (1.00-1.07)	7.40
Atrial fibrillation	Q1 (the lowest)	Reference	Reference	Reference	Reference
	Q2	1.03 (0.76-1.46)	1.04 (0.77-1.46)	0.99 (0.96-1.03)	NA
	Q3	1.22 (0.87-1.76)	1.23 (0.87-1.78)	0.99 (0.96-1.03)	NA
	Q4 (the highest)	1.68 (1.17-2.46)	1.60 (1.12-2.35)	1.05 (1.00-1.10)	11.21
Congestive heart failure	Q1 (the lowest)	Reference	Reference	Reference	Reference
	Q2	0.96 (0.70-1.38)	0.97 (0.71-1.36)	0.99 (0.95-1.04)	NA
	Q3	1.00 (0.70-1.40)	1.01 (0.72-1.40)	0.99 (0.94-1.04)	NA
	Q4 (the highest)	1.42 (0.99-2.01)	1.32 (0.93-1.87)	1.07 (1.00-1.15)	21.85

Values are HR (95% CI) unless otherwise indicated.

FGF = fibroblast growth factor.

a Mediation analysis models adjusted for age, sex, race and ethnicity, body mass index, current smoking, systolic blood pressure, history of coronary artery disease, congestive heart failure, stroke, peripheral vascular disease, diabetes, family history of coronary artery disease, estimated glomerular filtration rate, natural log-transformed urine albumin-to-creatinine ratio, serum albumin, hemoglobin, serum calcium, serum phosphate, natural log-transformed total parathyroid hormone, N-terminal pro-B-type natriuretic peptide, low-density lipoprotein, tumor necrosis factor α, fibrinogen, high sensitivity C-reactive protein, interleukin-6, interleukin-1β, and medication use (beta-blockers, angiotensin-converting enzyme inhibitors or angiotensin II receptor blockers, diuretics, aspirin, active vitamin D, phosphate binders, erythropoiesis stimulant, and statins).

b Proportion mediated was calculated by: DE × (IE−1)/(TE−1). Proportion mediated for Q2 and Q3 was not calculated because the point estimate for IE was null or negative.

**Central Illustration:**
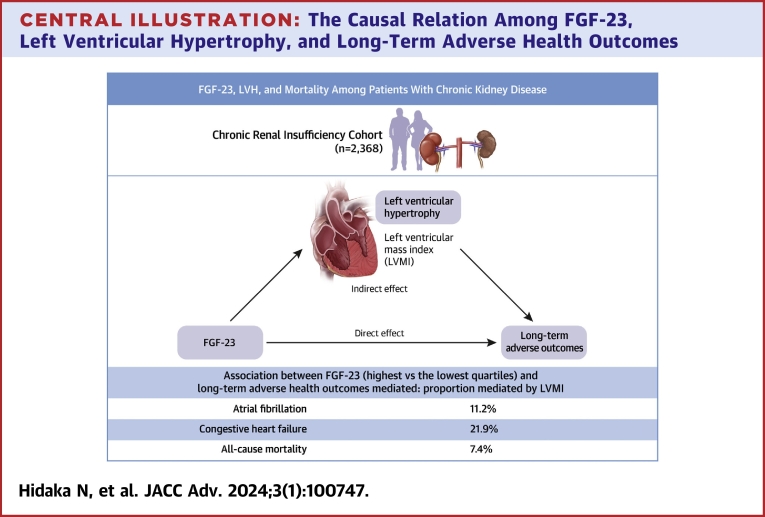
**The Causal Relation Among FGF-23, Left Ventricular Hypertrophy, and Long-Term Adverse Health Outcomes** We analyzed 2,368 participants enrolled in chronic renal insufficiency cohort study, the prospective observational cohort of chronic kidney disease patients. Fibroblast growth factor (FGF)-23 was associated with left ventricular mass index (LVMI) and increased risk of long-term adverse health outcomes (atrial fibrillation, congestive heart failure, and all-cause mortality). The proportion mediated (the proportion of indirect effect among the total effect) through left ventricular hypertrophy (LVH) was 7.4%, 11.2%, and 21.9% for all-cause mortality, atrial fibrillation, and congestive heart failure, respectively, suggesting that LVH had a relatively small role in the association between FGF-23 and long-term adverse health outcomes.

### Sensitivity analyses

The results did not qualitatively change when we: 1) analyzed the data without adjustment for the relevant biomarkers (Supplemental Tables 3 to 5); 2) included cystatin C-based eGFR instead of eGFR based on the MDRD equation in Model 3 (Supplemental Tables 3, 4, and 6); and 3) conducted mediation analysis using Model 2 instead of Model 3 (Supplemental Table 7). In addition, the analyses for only “definite” CHF, excluding probable CHF, were conducted, showing similar results (Supplemental Tables 8 and 9).

## Discussion

Using a prospective, multiracial cohort of CKD from the CRIC study spanning more than 10 years, we found an association between elevated C-term FGF-23 and an increase in LVMI or risks of long-term adverse health outcomes, as previously described.^9^^,^^10^^,^^24^^,^^25^ The estimated HRs among the highest quartile compared to the lowest quartile in our fully adjusted model were 1.62 for all-cause mortality, 1.58 for AF, and 1.32 for CHF. Our mediation analysis revealed that increased LVMI only partially mediated (7%-22%) the association between elevated C-term FGF-23 and these long-term adverse health outcomes (Central Illustration). These results indicate the potential overestimation of the hypothesis that FGF-23 itself induces LVH, leading to a high incidence of death or cardiovascular events among CKD patients.

When we used the same covariates as previously reported by Isacova et al^9^ in 2011 in our data with a median follow-up of 12.0 years, we found a 1.71-fold risk of all-cause death in the highest quartile of C-term FGF-23 compared to the lowest quartile, which was smaller than the 3.0-fold risk in the previous study with a median follow-up of 3.5 years. Higher risks of incident AF and CHF according to the increase in C-term FGF-23 level were also observed in the current analysis, and the HRs (ie, 1.66 for AF and 1.37 for CHF) were comparable or smaller than in the prior studies.^24^^,^^25^ Given such differences in follow-up periods and estimates between our results and previous findings, although these cohorts were not completely identical to each other, whether the effect of C-term FGF-23 on long-term adverse health outcomes varies in a time-dependent manner should be the subject of future research.

Our study further adjusted for the covariates recently identified as the modifier of C-term FGF-23, including the biomarkers of inflammation and the use of erythropoiesis-stimulating agents. In addition, a family history of coronary artery disease, NT-proBNP, and the use of diuretics, active vitamin D, and phosphate binders, which might be associated with C-term FGF-23, LVH, or cardiovascular outcomes, were additionally adjusted in Model 3.^28^^,^^36^ Compared to Model 2, the magnitude of the associations of C-term FGF-23 with LVMI and long-term adverse health outcomes decreased after adjusting for these newly added variables in Model 3. While the current analysis could not employ the data relevant to iron status (eg, serum iron, ferritin, and total iron binding capacity), iron deficiency was confirmed to be one of the modifiers of C-term FGF-23.^17^^,^^18^ Considering the high prevalence of iron deficiency among CKD patients, additional adjustment with iron status data might further water down the association between C-term FGF-23 and LVH, all-cause mortality, and cardiovascular outcomes.

To the best of our knowledge, this is the first study to quantify the extent to which LVMI contributes to the association between C-term FGF-23 and long-term adverse health outcomes. Our findings that approximately 10% of the association of C-term FGF-23 with all-cause mortality and incident AF was mediated by LVMI may be explained by the following 3 possibilities. First, some factors other than LVH, such as atherosclerosis,^37^ hypertension, or impaired leukocyte recruitment,^38^ mediate the association between C-term FGF-23 and long-term adverse health outcomes. Second, the trajectory of C-term FGF-23 might be important for the development of LVH, and thus, a single measurement of C-term FGF-23 may not be sufficient.^39^ Last, the observed small estimated indirect effect might reflect that the effect of C-term FGF-23 on LVH leading to long-term adverse health outcomes might be small in humans. The direct effect of FGF-23 on the induction of LVH and the protective action of FGF receptor blockade have been validated not in humans but only in in vitro and in vivo animal settings.^10^^,^^40^, ^41^, ^42^ To determine the role of FGF-23 in the development of long-term adverse health outcomes, trials to evaluate the effect of FGF receptor blockade on preventing long-term adverse health outcomes in CKD model animals or patients are ideally required. Taken together, the current results suggest that studies on the effect of FGF-23 on LVH are rather misdirected in seeking a novel treatment option to improve the prognosis of patients with CKD. In addition, the associations observed between FGF-23 and LVH, or long-term adverse health outcomes might not reflect a causal relation and might be a result of unadjusted confounding bias.

### Study limitations

First, echocardiography data only at 1 year after the baseline visit were included to avoid the potential competing risk for the mediator (LVMI) by early occurrence of cardiovascular outcomes and death. In a previous report, 20% of CKD patients without LVH developed new-onset LVH within a median of 2.9 years.^10^ Therefore, we could not fully capture the incidence of LVH thereafter during the follow-up, which might underestimate the mediation of LVMI in the association between FGF-23 and cardiovascular outcomes. Second, our findings suffered from unmeasured confounders. In mediation analysis, we assume there are no unmeasured confounders in all 3 pathways (ie, exposure-mediator, exposure-outcome, and mediator-outcome pathways). However, for example, iron status data at baseline were not available. This was considered to be one of the most influencing confounders affecting the serum levels of C-term FGF-23 because iron deficiency is an established factor that modifies the amount of C-term FGF-23.^17^^,^^18^ Third, the changes in standards of medical care for CKD patients within a follow-up period, such as the changes in recommendations for control of blood pressure, blood glucose, or lipid profile, and the development of new drugs that might improve the prognosis of CKD patients with heart failure (eg, sodium-glucose cotransporter-2 inhibitors and glucagon-like peptide-1 receptor agonists), could decrease the incidence of outcomes. Last, we cannot rule out the possibility of selection bias given that our study included CRIC participants with available data on C-term FGF-23 at baseline, echocardiography at 1 year, and outcomes.

## Conclusions

Using an established, multiracial, and prospective CKD cohort, we extended the observational period and found associations of elevated C-term FGF-23 with increased LVMI and increased risks of all-cause mortality, incident AF, and incident CHF. Our quantitative mediation analysis revealed that increased LVMI mediated a small part of the hypothetical causal pathway from C-term FGF-23 to all-cause mortality and incident AF: 7.40% and 11.21%, respectively (Central Illustration). Although these results would not contradict the hypothesis that FGF-23 directly stimulates cardiomyocytes and induces LVH, resulting in death or cardiovascular events among CKD patients, the current study revealed that the contribution of the development of LVH played a lesser role in this pathway than previously expected.

PERSPECTIVES **COMPETENCY IN MEDICAL KNOWLEDGE:** FGF-23, which elevates secondarily in CKD patients, is a hormone to regulate phosphate homeostasis. Recently, some populational studies for CKD patients reported the association between elevated FGF-23 and high incidence of cardiovascular disease or all-cause death, and some studies with animal model of CKD suggested that FGF-23 itself induces LVH, which might lead to cardiovascular comorbidities and mortality. The current study with a causal mediation analysis could quantify the extent to which LVH mediates the effect of FGF-23 on cardiovascular disease or all-cause death in a CKD cohort, showing that only 7.4%, 11.2%, and 21.9% of the total effects of FGF-23 were mediated by LVH for all-cause mortality, atrial fibrillation, and congestive heart failure, respectively.

**TRANSLATIONAL OUTLOOK:** Additional research to clarify other potential mediators is required to determine the role of FGF-23 in the long-term adverse health outcomes in CKD patients because the mediational effect of LVH was much smaller than expected. Furthermore, the other possibility should always be considered that FGF-23 is a mere bystander that just correlates with LVH, cardiovascular disease, or all-cause mortality and does not directly affect the prognosis of CKD patients.

## Funding support and author disclosures

Dr Nangaku has received research support and honoraria from 10.13039/501100004095Kyowa Kirin Co, Ltd. Dr Ito has received research support from 10.13039/501100004095Kyowa Kirin Co, Ltd. All other authors have reported that they have no relationships relevant to the contents of this paper to disclose.
