# Supplementary material for: FGF-23, Left Ventricular Hypertrophy, and Mortality in Patients With CKD: A Revisit With Mediation Analysis
Source: JACC Adv. 2023 Dec 9;3(1):100747. doi: 10.1016/j.jacadv.2023.100747 (PMC11198284; doi:10.1016/j.jacadv.2023.100747)
Supplement: Supplemental Methods and Supplemental Tables 1-9 [file mmc1.docx]

**Supplemental Methods:** Causal mediation analysis in this study

In this study, we applied causal mediation analysis to estimate the natural direct effect and natural indirect effect (mediated through left ventricular hypertrophy) of FGF23 on all-cause mortality and cardiovascular outcomes. In our mediation analysis with the g-formula approach (R package “CMAverse”), we used (i) the linear regression model for the mediator model, and (ii) the Cox proportional hazard regression model for the outcome model. The robust 95% confidence intervals were obtained from 1000 bootstrapped samples.


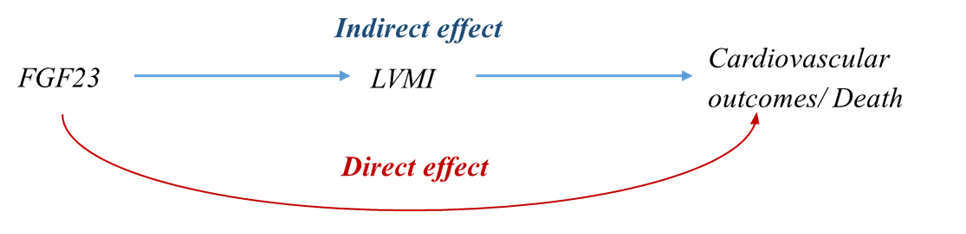


To obtain unbiased estimates from causal mediation analysis, we need the following assumptions: 1) the causal path from the exposure (FGF23) to the outcome (all-cause death or cardiovascular outcomes) is not confounded given the measured covariates, 2) the causal path from the exposure (FGF23) to the mediator (LVMI) is not confounded given the measured covariates, 3) the causal path from the mediator (LVMI) to the outcome (all-cause death or cardiovascular outcomes) is not confounded given the measured covariates, and 4) there is no confounders between the mediator (LVMI) and the outcome (all-cause death or cardiovascular outcomes) affected by the exposure (FGF23). In addition, we need other causal modeling assumptions including consistency, composition, well-defined variables, no model misspecification, and no other sources of bias.

Through this approach under the above-mentioned assumptions, we can quantify the extent to which left ventricular hypertrophy mediates the pathway from FGF23 to health outcomes. The directed acyclic digraph under investigation of this study is shown below. In our main analysis, we included all these variables. However, because we could not determine the temporality between FGF23 and variables that interact with FGF23 (i.e., serum calcium, serum phosphate, total parathyroid hormone, and use of active vitamin D and phosphate binders), we also conducted the sensitivity analysis excluding these variables.

**Directed acyclic graph under investigation**


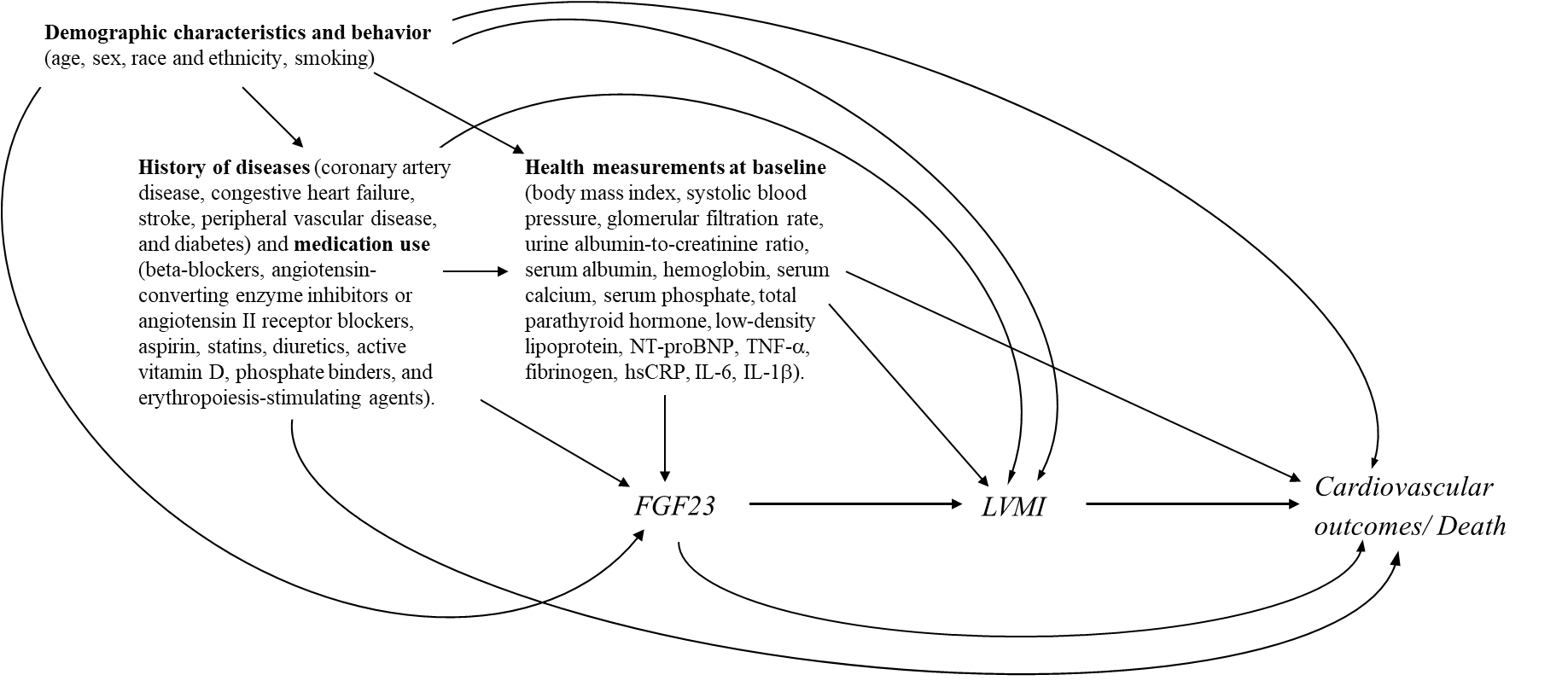


**Supplemental Table 1**. Comparison of the baseline characteristics among 2610 participants with baseline C-terminal FGF23 and LVMI at one-year, the final analytical cohort (2368 participants), and the excluded 242 participants

|  | Cohort before exclusion | Final analytical cohort | Excluded participants |
| --- | --- | --- | --- |
| Total, n ^a^ | 2610 | 2368 | 242 |
| Characteristics, n (%) |  |  |  |
| Age, years | 57.6 (10.8) | 57.7 (10.7) | 56.0 (11.6) |
| Male | 1390 (53.3) | 1252 (52.9) | 138 (57.0) |
| Race |  |  |  |
| White | 1189 (45.6) | 1093 (46.2) | 96 (39.7) |
| Black | 1183 (45.3) | 1054 (44.5) | 129 (53.3) |
| Hispanic | 125 (4.8) | 117 (4.9) | 8 (3.3) |
| Others | 113 (4.3) | 104 (4.4) | 9 (3.7) |
| Body mass index, kg/m^2^ | 31.6 (7.3) | 31.7 (7.3) | 31.1 (6.7) |
| Current smoker | 328 (12.6) | 283 (12.0) | 45 (18.6) |
| Systolic blood pressure, mmHg | 127.1 (21.3) | 126.7 (21.1) | 130.8 (23.2) |
| Past history |  |  |  |
| Coronary artery disease | 546 (20.9) | 493 (20.8) | 53 (21.9) |
| Congestive heart failure | 233 (8.9) | 212 (9.0) | 21 (8.7) |
| Stroke | 246 (9.4) | 220 (9.3) | 26 (10.7) |
| Peripheral vascular disease | 164 (6.3) | 142 (6.0) | 22 (9.1) |
| Diabetes | 1186 (45.4) | 1068 (45.1) | 118 (48.8) |
| Family history of coronary artery disease | 397 (15.2) | 352 (14.9) | 45 (18.6) |
| Laboratory results |  |  |  |
| Estimated glomerular filtration rate, mL/min/1.73m^2^ | 43.5 (13.3) | 43.5 (13.2) | 44.0 (14.1) |
| Urine albumin-to-creatinine ratio, mg/g | 522.3 (1410.1) | 509.9 (1376.5) | 700.3 (1819.9) |
| Serum albumin, g/dL | 4.0 (0.5) | 4.0 (0.4) | 3.9 (0.5) |
| Hemoglobin, g/dL | 12.7 (1.8) | 12.7 (1.8) | 12.8 (1.9) |

**Supplemental Table 1.** Comparison of the baseline characteristics among 2610 participants with baseline C-term FGF23 and LVMI at one-year, the final analytical cohort (2368 participants), and the excluded 242 participants (continued)

|  | Cohort before exclusion | Final analytical cohort | Excluded participants |
| --- | --- | --- | --- |
| Total, n | 2610 | 2368 | 242 |
| Calcium, mg/dL | 9.2 (0.5) | 9.2 (0.5) | 9.2 (0.5) |
| Phosphate, mg/dL | 3.7 (0.7) | 3.7 (0.7) | 3.8 (0.7) |
| Total parathyroid hormone, pg/mL | 71.8 (68.7) | 71.5 (67.2) | 76.2 (85.3) |
| N-terminal pro-B-type natriuretic peptide, pg/mL | 451.2 (1577.9) | 440.8 (1484.1) | 579.1 (2457.9) |
| LDL, mg/dL | 102 (35) | 102 (35) | 105 (37) |
| Tumor necrosis factor-α, pg/mL | 3.1 (12.1) | 3.1 (12.6) | 2.7 (2.7) |
| Fibrinogen, g/L | 4.1 (1.2) | 4.1 (1.2) | 4.2 (1.2) |
| High sensitivity C-reactive protein, mg/L | 5.4 (9.7) | 5.3 (9.6) | 5.8 (10.5) |
| Interleukin-6, pg/mL | 4.1 (17.6) | 4.1 (17.8) | 4.4 (15.4) |
| Interleukin-1β, pg/mL | 1.1 (3.2) | 1.1 (3.3) | 1.0 (2.0) |
| Medication use |  |  |  |
| Beta-blockers | 1255 (48.1) | 1132 (47.8) | 123 (50.8) |
| Angiotensin-converting enzyme inhibitors or Angiotensin-II receptor blockers | 1781 (68.2) | 1608 (67.9) | 173 (71.5) |
| Diuretics | 1513 (58.0) | 1363 (57.6) | 150 (62.0) |
| Aspirin | 1146 (43.9) | 1037 (43.8) | 109 (45.0) |
| Active vitamin D | 87 (3.3) | 75 (3.2) | 12 (5.0) |
| Phosphate binders | 180 (6.9) | 161 (6.8) | 19 (7.9) |
| Erythropoiesis-stimulating agents | 103 (3.9) | 96 (4.1) | 7 (2.9) |
| Statins | 1462 (56.0) | 1334 (56.3) | 128 (52.9) |

^a^Numerical variables are described as the mean (standard deviation). FGF23: Fibroblast growth factor 23; LVMI: Left ventricular mass index

**Supplemental Table 2**. Baseline C-term FGF23 levels and incidence of atrial fibrillation or congestive heart failure using competing-risks regression based on Fine and Gray's proportional subhazards model.

| **Events** | **Quartiles of FGF23** | **Unadjusted** | | **Model 1**^a^ | | **Model 2**^b^ | | **Model 3**^c^ | | |
| --- | --- | --- | --- | --- | --- | --- | --- | --- | --- | --- |
|  |  | **HR** | **95% CI** | **HR** | **95% CI** | **HR** | **95% CI** | **HR** | | **95% CI** |
| Atrial fibrillation | Q1 (the lowest) | Reference | | Reference | | Reference | | Reference | | |
|  | Q2 | 1.24 | 0.92-1.69 | 1.13 | 0.83-1.54 | 1.02 | 0.74-1.42 | 1.02 | 0.74-1.41 | |
|  | Q3 | 1.54 | 1.15-2.07 | 1.45 | 1.07-1.95 | 1.17 | 0.84-1.63 | 1.16 | 0.83-1.62 | |
|  | Q4 (the highest) | 2.07 | 1.56-2.74 | 2.15 | 1.60-2.88 | 1.54 | 1.08-2.20 | 1.45 | 1.01-2.07 | |
|  | *P for trend* | < 0.001 | | < 0.001 | | 0.018 | | 0.044 | | |
| Congestive heart failure | Q1 (the lowest) | Reference | | Reference | | Reference | | Reference | | |
|  | Q2 | 1.48 | 1.09-2.01 | 1.37 | 1.01-1.86 | 0.97 | 0.71-1.33 | 0.95 | 0.69-1.31 | |
|  | Q3 | 2.11 | 1.59-2.82 | 1.96 | 1.47-2.63 | 0.98 | 0.71-1.35 | 0.95 | 0.69-1.32 | |
|  | Q4 (the highest) | 3.80 | 2.89-4.99 | 3.58 | 2.71-4.73 | 1.24 | 0.88-1.73 | 1.19 | 0.85-1.67 | |
|  | *P for trend* | < 0.001 | | < 0.001 | | 0.22 | | 0.31 | | |

^a^Adjusted for age, sex, race and ethnicity.

^b^Adjusted for age, sex, race and ethnicity, body mass index, current smoking, systolic blood pressure, history of coronary artery disease, congestive heart failure, stroke, peripheral vascular disease, and diabetes, estimated glomerular filtration rate, natural log-transformed urine albumin-to-creatinine ratio, serum albumin, hemoglobin, serum calcium, serum phosphate, natural log-transformed total parathyroid hormone, low-density lipoprotein, and medication use (beta-blockers, angiotensin-converting enzyme inhibitors or angiotensin II receptor blockers, aspirin, and statins).

^c^Adjusted for covariates in Model 2 plus family history of coronary artery disease, N-terminal pro-B-type natriuretic peptide, tumor necrosis factor α, fibrinogen, high sensitivity C-reactive protein, interleukin-6, interleukin-1β, and medication use (diuretics, active vitamin D, phosphate binders, and erythropoiesis-stimulating agents).

FGF23: Fibroblast growth factor 23

**Supplemental Table 3.** Baseline C-term FGF23 levels and left ventricular hypertrophy at the 1-year follow-up with Model 4^a^ and Model 5^b^

| ***log (LVMI)*** | **Model 4** ^a^ | | **Model 5** ^b^ | |
| --- | --- | --- | --- | --- |
|  | **β×10^2^** | **95% CI** | **β×10^2^** | **95% CI** |
| ***Quartiles of FGF23*** |  |  |  |  |
| Q1 (the lowest) | Reference | | Reference | |
| Q2 | -0.13 | -2.51 to 2.26 | -0.38 | -2.79 to 2.02 |
| Q3 | -0.37 | -2.91 to 2.18 | -0.50 | -3.09 to 2.08 |
| Q4 (the highest) | 3.65 | 0.78 to 6.53 | 3.26 | 0.32 to 6.21 |
| *P for trend* | 0.013 | | 0.030 | |

^a^Adjusted for the covariates in Model 3 excluding several variables that interact with FGF23 (serum calcium, serum phosphate, total parathyroid hormone, and use of active vitamin D and phosphate binders). That is, Model 4 adjusted for age, sex, race and ethnicity, body mass index, current smoking, systolic blood pressure, history of coronary artery disease, congestive heart failure, stroke, peripheral vascular disease, diabetes, family history of coronary artery disease, estimated glomerular filtration rate, natural log-transformed urine albumin-to-creatinine ratio, serum albumin, hemoglobin, low-density lipoprotein, N-terminal pro-B-type natriuretic peptide, tumor necrosis factor α, fibrinogen, high sensitivity C-reactive protein, interleukin-6, interleukin-1β, and medication use (beta-blockers, angiotensin-converting enzyme inhibitors or angiotensin II receptor blockers, aspirin, statins, diuretics, and erythropoiesis-stimulating agents).

^b^Adjusted for the covariates in Model 3, but the estimated glomerular filtration rate was based on serum cystatin C.

FGF23: Fibroblast growth factor 23; LVMI: Left ventricular mass index**Supplemental Table 4.** Baseline C-term FGF23 levels and all-cause mortality, incidence of atrial fibrillation or congestive heart failure, with Model 4^a^ and Model 5^b^

| **Events** | **Quartiles of FGF23** | **Model 4** ^a^ | | **Model 5** ^b^ | |
| --- | --- | --- | --- | --- | --- |
|  |  | **HR** | **95% CI** | **HR** | **95% CI** |
| All-cause mortality | Q1 (the lowest) | Reference | | Reference | |
|  | Q2 | 0.91 | 0.70-1.17 | 0.84 | 0.65-1.09 |
|  | Q3 | 1.30 | 1.01-1.66 | 1.17 | 0.91-1.51 |
|  | Q4 (the highest) | 1.62 | 1.25-2.11 | 1.41 | 1.08-1.84 |
|  | *P for trend* | < 0.001 | | 0.011 | |
| Atrial fibrillation | Q1 (the lowest) | Reference | | Reference | |
|  | Q2 | 1.02 | 0.74-1.40 | 0.98 | 0.71-1.35 |
|  | Q3 | 1.17 | 0.85-1.61 | 1.10 | 0.79-1.52 |
|  | Q4 (the highest) | 1.54 | 1.10-2.17 | 1.42 | 1.00-2.02 |
|  | *P for trend* | 0.012 | | 0.049 | |
| Congestive heart failure | Q1 (the lowest) | Reference | | Reference | |
|  | Q2 | 0.99 | 0.72-1.36 | 0.96 | 0.70-1.32 |
|  | Q3 | 0.98 | 0.71-1.34 | 0.94 | 0.68-1.30 |
|  | Q4 (the highest) | 1.32 | 0.96-1.83 | 1.25 | 0.89-1.74 |
|  | *P for trend* | 0.091 | | 0.194 | |

^a^Adjusted for the covariates in Model 3 excluding several variables that interact with FGF23 (serum calcium, serum phosphate, total parathyroid hormone, and use of active vitamin D and phosphate binders). That is, Model 4 adjusted for age, sex, race and ethnicity, body mass index, current smoking, systolic blood pressure, history of coronary artery disease, congestive heart failure, stroke, peripheral vascular disease, diabetes, family history of coronary artery disease, estimated glomerular filtration rate, natural log-transformed urine albumin-to-creatinine ratio, serum albumin, hemoglobin, low-density lipoprotein, N-terminal pro-B-type natriuretic peptide, tumor necrosis factor α, fibrinogen, high sensitivity C-reactive protein, interleukin-6, interleukin-1β, and medication use (beta-blockers, angiotensin-converting enzyme inhibitors or angiotensin II receptor blockers, aspirin, statins, diuretics, and erythropoiesis-stimulating agents).

^b^Adjusted for the covariates in Model 3, but the estimated glomerular filtration rate was based on serum cystatin C.

FGF23: Fibroblast growth factor 23

**Supplemental Table 5.** Direct and indirect effects (hazard ratio scale [95% CI]) of FGF23 levels on all-cause mortality and the incidence of atrial fibrillation or congestive heart failure via left ventricular hypertrophy, using the model excluding several variables that interact with C-term FGF23 (serum calcium, serum phosphate, total parathyroid hormone, and use of active vitamin D and phosphate binders)^a^

| **Events** | **Quartiles of FGF23** | **Total effect (TE)** | **Direct effect (DE)** | **Indirect effect (IE)** | **%mediated**^b^ |
| --- | --- | --- | --- | --- | --- |
|  |  | **HR (95% CI)** | **HR (95% CI)** | **HR (95% CI)** |  |
| All-cause mortality | Q1 (the lowest) | Reference | Reference | Reference | Reference |
|  | Q2 | 0.89 (0.69-1.15) | 0.89 (0.69-1.15) | 1.00 (0.98-1.02) | NA |
|  | Q3 | 1.28 (0.98-1.66) | 1.29 (0.98-1.66) | 1.00 (0.97-1.02) | NA |
|  | Q4 (the highest) | 1.69 (1.29-2.23) | 1.63 (1.24-2.17) | 1.04 (1.01-1.07) | 8.46 |
| Atrial fibrillation | Q1 (the lowest) | Reference | Reference | Reference | Reference |
|  | Q2 | 1.02 (0.74-1.42) | 1.02 (0.75-1.41) | 1.00 (0.97-1.03) | NA |
|  | Q3 | 1.18 (0.85-1.71) | 1.19 (0.84-1.73) | 0.99 (0.96-1.03) | NA |
|  | Q4 (the highest) | 1.61 (1.13-2.33) | 1.52 (1.08-2.22) | 1.05 (1.01-1.11) | 13.60 |
| Congestive heart failure | Q1 (the lowest) | Reference | Reference | Reference | Reference |
|  | Q2 | 0.96 (0.70-1.38) | 0.96 (0.70-1.37) | 1.00 (0.95-1.05) | NA |
|  | Q3 | 0.99 (0.70-1.38) | 0.99 (0.71-1.37) | 0.99 (0.94-1.04) | NA |
|  | Q4 (the highest) | 1.42 (1.01-2.01) | 1.31 (0.93-1.83) | 1.08 (1.01-1.16) | 25.47 |

^a^Mediation analysis models adjusted for age, sex, race and ethnicity, body mass index, current smoking, systolic blood pressure, history of coronary artery disease, congestive heart failure, stroke, peripheral vascular disease, and diabetes, family history of coronary artery disease, estimated glomerular filtration rate, serum urea nitrogen, urine protein level, hemoglobin, N-terminal pro-B-type natriuric peptide, low-density lipoprotein, tumor necrosis factor α, fibrinogen, high sensitivity CRP, interleukin-6, interleukin-1β, and medication use (beta-blockers, angiotensin-converting enzyme inhibitors or angiotensin II receptor blockers, diuretics, aspirin, erythropoiesis-stimulating agents, and statins).

^b^ Proportion mediated was calculated by DE×(IE-1)/(TE-1). Proportion mediated for Q2 and Q3 was not calculated because the point estimate for IE was null or negative.

FGF23: Fibroblast growth factor 23

**Supplemental Table 6.** Direct and indirect effects (hazard ratio scale [95% CI]) of C-term FGF23 levels on all-cause mortality and the incidence of atrial fibrillation or congestive heart failure via left ventricular hypertrophy, using the model with estimated glomerular filtration rate based on cystatin C^a^

| **Events** | **Quartiles of FGF23** | **Total effect (TE)** | **Direct effect (DE)** | **Indirect effect (IE)** | **%mediated**^b^ |
| --- | --- | --- | --- | --- | --- |
|  |  | **HR (95% CI)** | **HR (95% CI)** | **HR (95% CI)** |  |
| All-cause mortality | Q1 (the lowest) | Reference | Reference | Reference | Reference |
|  | Q2 | 0.82 (0.64-1.07) | 0.82 (0.64-1.07) | 1.00 (0.97-1.02) | NA |
|  | Q3 | 1.14 (0.86-1.48) | 1.14 (0.87-1.48) | 1.00 (0.97-1.02) | NA |
|  | Q4 (the highest) | 1.43 (1.08-1.92) | 1.39 (1.04-1.88) | 1.03 (1.00-1.07) | 10.02 |
| Atrial fibrillation | Q1 (the lowest) | Reference | Reference | Reference | Reference |
|  | Q2 | 0.98 (0.71-1.41) | 0.99 (0.72-1.40) | 0.99 (0.96-1.03) | NA |
|  | Q3 | 1.11 (0.79-1.62) | 1.11 (0.80-1.63) | 0.99 (0.96-1.03) | NA |
|  | Q4 (the highest) | 1.47 (1.03-2.18) | 1.40 (0.99-2.07) | 1.05 (1.00-1.10) | 14.09 |
| Congestive heart failure | Q1 (the lowest) | Reference | Reference | Reference | Reference |
|  | Q2 | 0.92 (0.67-1.32) | 0.93 (0.68-1.31) | 0.99 (0.95-1.04) | NA |
|  | Q3 | 0.94 (0.66-1.31) | 0.96 (0.67-1.31) | 0.99 (0.94-1.04) | NA |
|  | Q4 (the highest) | 1.30 (0.91-1.86) | 1.22 (0.84-1.73) | 1.07 (1.00-1.15) | 25.77 |

^a^Mediation analysis models adjusted for age, sex, race and ethnicity, body mass index, current smoking, systolic blood pressure, history of coronary artery disease, congestive heart failure, stroke, peripheral vascular disease, diabetes, family history of coronary artery disease, estimated glomerular filtration rate based on cystatin C, serum urea nitrogen, urine protein level, hemoglobin, N-terminal pro-B-type natriuric peptide, low-density lipoprotein, tumor necrosis factor α, fibrinogen, high sensitivity CRP, interleukin-6, interleukin-1β, and medication use (beta-blockers, angiotensin-converting enzyme inhibitors or angiotensin II receptor blockers, diuretics, aspirin, erythropoiesis-stimulating agents, and statins).

^b^ Proportion mediated was calculated by DE×(IE-1)/(TE-1). Proportion mediated for Q2 and Q3 was not calculated because the point estimate for IE was null or negative.

FGF23: Fibroblast growth factor 23

**Supplemental Table 7.** Direct and indirect effects (hazard ratio scale [95% CI]) of C-term FGF23 levels on all-cause mortality and the incidence of atrial fibrillation or congestive heart failure via left ventricular hypertrophy, using Model 2^a^

| **Events** | **Quartiles of FGF23** | **Total effect (TE)** | **Direct effect (DE)** | **Indirect effect (IE)** | **%mediated**^b^ |
| --- | --- | --- | --- | --- | --- |
|  |  | **HR (95% CI)** | **HR (95% CI)** | **HR (95% CI)** |  |
| All-cause mortality | Q1 (the lowest) | Reference | Reference | Reference | Reference |
|  | Q2 | 0.90 (0.69-1.14) | 0.90 (0.69-1.15) | 1.00 (0.97-1.02) | NA |
|  | Q3 | 1.29 (0.99-1.67) | 1.30 (0.99-1.66) | 1.00 (0.97-1.02) | NA |
|  | Q4 (the highest) | 1.80 (1.35-2.40) | 1.73 (1.30-2.31) | 1.04 (1.01-1.08) | 8.81 |
| Atrial fibrillation | Q1 (the lowest) | Reference | Reference | Reference | Reference |
|  | Q2 | 1.03 (0.74-1.44) | 1.03 (0.75-1.43) | 1.00 (0.96-1.04) | NA |
|  | Q3 | 1.23 (0.88-1.75) | 1.23 (0.88-1.76) | 0.99 (0.95-1.03) | NA |
|  | Q4 (the highest) | 1.79 (1.23-2.62) | 1.68 (1.16-2.46) | 1.06 (1.01-1.12) | 13.16 |
| Congestive heart failure | Q1 (the lowest) | Reference | Reference | Reference | Reference |
|  | Q2 | 0.97 (0.71-1.39) | 0.97 (0.71-1.36) | 1.00 (0.95-1.05) | NA |
|  | Q3 | 1.03 (0.73-1.40) | 1.03 (0.75-1.40) | 0.99 (0.94-1.04) | NA |
|  | Q4 (the highest) | 1.49 (1.05-2.10) | 1.37 (0.99-1.94) | 1.09 (1.01-1.17) | 24.22 |

^a^Mediation analysis models adjusted for age, sex, race and ethnicity, body mass index, current smoking, systolic blood pressure, history of coronary artery disease, congestive heart failure, stroke, peripheral vascular disease, diabetes, estimated glomerular filtration rate, natural log-transformed urine albumin-to-creatinine ratio, serum albumin, hemoglobin, serum calcium, serum phosphate, natural log-transformed total parathyroid hormone, low-density lipoprotein, and medication use (beta-blockers, angiotensin-converting enzyme inhibitors or angiotensin II receptor blockers, aspirin, and statins).

^b^ Proportion mediated was calculated by DE×(IE-1)/(TE-1). Proportion mediated for Q2 and Q3 was not calculated because the point estimate for IE was null or negative.

FGF23: Fibroblast growth factor 23

**Supplemental Table 8.** Baseline C-term FGF23 levels and incidence of definite congestive heart failure

| **Events** | **Quartiles of FGF23** | **Unadjusted** | | **Model 1**^a^ | | **Model 2**^b^ | | **Model 3**^c^ | | |
| --- | --- | --- | --- | --- | --- | --- | --- | --- | --- | --- |
|  |  | **HR** | **95% CI** | **HR** | **95% CI** | **HR** | **95% CI** | **HR** | | **95% CI** |
| Definite  congestive heart failure | Q1 (the lowest) | Reference | | Reference | | Reference | | Reference | | |
|  | Q2 | 1.52 | 1.07-2.14 | 1.39 | 0.98-1.97 | 1.02 | 0.71-1.45 | 1.01 | 0.70-1.44 | |
|  | Q3 | 2.04 | 1.46-2.84 | 1.91 | 1.37-2.68 | 0.97 | 0.68-1.39 | 0.94 | 0.65-1.35 | |
|  | Q4 (the highest) | 4.22 | 3.11-5.74 | 4.13 | 3.02-5.65 | 1.41 | 0.97-2.04 | 1.35 | 0.93-1.97 | |
|  | *P for trend* | < 0.001 | | < 0.001 | | 0.070 | | 0.111 | | |

^a^Adjusted for age, sex, race and ethnicity.

^b^Adjusted for age, sex, race and ethnicity, body mass index, current smoking, systolic blood pressure, history of coronary artery disease, congestive heart failure, stroke, peripheral vascular disease, and diabetes, estimated glomerular filtration rate, natural log-transformed urine albumin-to-creatinine ratio, serum albumin, hemoglobin, serum calcium, serum phosphate, natural log-transformed total parathyroid hormone, low-density lipoprotein, and medication use (beta-blockers, angiotensin-converting enzyme inhibitors or angiotensin II receptor blockers, aspirin, and statins).

^c^Adjusted for covariates in Model 2 plus family history of coronary artery disease, N-terminal pro-B-type natriuretic peptide, tumor necrosis factor α, fibrinogen, high sensitivity C-reactive protein, interleukin-6, interleukin-1β, and medication use (diuretics, active vitamin D, phosphate binders, and erythropoiesis-stimulating agents).

FGF23: Fibroblast growth factor 23

**Supplemental Table 9.** Direct and indirect effects (hazard ratio scale [95% CI]) of C-term FGF23 levels on the incidence of definite congestive heart failure via left ventricular hypertrophy^a^

| **Events** | **Quartiles of FGF23** | **Total effect (TE)** | **Direct effect (DE)** | **Indirect effect (IE)** | **%mediated**^b^ |
| --- | --- | --- | --- | --- | --- |
|  |  | **HR (95% CI)** | **HR (95% CI)** | **HR (95% CI)** |  |
| Definite  congestive heart failure | Q1 (the lowest) | Reference | Reference | Reference | Reference |
|  | Q2 | 0.96 (0.69-1.45) | 0.97 (0.68-1.43) | 0.99 (0.95-1.04) | NA |
|  | Q3 | 0.94 (0.62-1.40) | 0.95 (0.65-1.41) | 0.99 (0.94-1.04) | NA |
|  | Q4 (the highest) | 1.43 (0.96-2.19) | 1.34 (0.89-2.04) | 1.07 (1.00-1.16) | 21.13 |

^a^Mediation analysis models adjusted for age, sex, race and ethnicity, body mass index, current smoking, systolic blood pressure, history of coronary artery disease, congestive heart failure, stroke, peripheral vascular disease, diabetes, family history of coronary artery disease, estimated glomerular filtration rate, natural log-transformed urine albumin-to-creatinine ratio, serum albumin, hemoglobin, serum calcium, serum phosphate, natural log-transformed total parathyroid hormone, N-terminal pro-B-type natriuretic peptide, low-density lipoprotein, tumor necrosis factor α, fibrinogen, high sensitivity C-reactive protein, interleukin-6, interleukin-1β, and medication use (beta-blockers, angiotensin-converting enzyme inhibitors or angiotensin II receptor blockers, diuretics, aspirin, active vitamin D, phosphate binders, erythropoiesis stimulant, and statins).

^b^Proportion mediated was calculated by DE×(IE-1)/(TE-1). Proportion mediated for Q2 and Q3 was not calculated because the point estimate for IE was null or negative.

FGF23: Fibroblast growth factor 23
